# Acute necrotizing pneumonia combined with parapneumonic effusion caused by *Mycobacterium lentiflavum*: a case report

**DOI:** 10.1186/s12879-015-1100-z

**Published:** 2015-08-19

**Authors:** Yong Chul Lee, Seung Bum Kim, Su Jin Gang, Seung Yong Park, So Ri Kim

**Affiliations:** Department of Internal Medicine, Research Center for Pulmonary Disorders, Chonbuk National University Medical School, Research Institute of Clinical Medicine of Chonbuk National University-Biomedical Research Institute of Chonbuk National University Hospital, Jeonju, South Korea

**Keywords:** *Mycobacterium lentiflavum*, Nontuberculous mycobacterium, Pleural effusion, Necrotizing pneumonia

## Abstract

**Background:**

*Mycobacterium lentiflavum* (*M. lentiflavum*), a slow growing nontuberculous mycobacterium (NTM), has recently been described as an emerging human pathogen regardless of the immune status of the host. Previous reports have demonstrated that cervical lymphadenitis of children is the most frequent pathology of *M. lentiflavum*. However, there are little reports regarding pulmonary diseases by *M. lentiflavum* specifically in immunocompetent patients.

**Case presentation:**

A 60-year-old man having prolonged productive cough and dyspnea with fever was initially diagnosed as pneumonia with parapneumonic effusion. Imaging studies showed that the radiologic abnormality was acute bronchopneumonic infiltration with abscess formation in the left lower lobe and parapneumonic pleural effusion. *M. lentiflavum* was identified in the cultured pleural tissues. On the basis of these findings, he was diagnosed as pulmonary infection and pleurisy caused by *M. lentiflavum*, which was treated with a combination of antibiotics covering NTM. His clinical manifestations were dramatically improved by the treatment targeting NTM, while those were refractory to empirical antibiotic therapy.

**Conclusion:**

In this report, we introduce the isolation of *M. lentiflavum* from pleural tissues associated with acute necrotizing pneumonia combined with parapneumonic effusion in an immunocompetent host, suggesting that the *M. lentiflavum* can be a human pathogen invovled in pulmonary infectious diseases and pleurisy with poor response to empirical antibiotic treatment.

## Background

*Mycobacterium lentiflavum* (*M. lentiflavum*), one of slowly growing nontuberculous mycobacterium (NTM), was first identified in 1996 as a distinct strain [1]. This strain is characterized by slow growth of tiny yellow colonies and has biochemical characteristics identical to the *Mycobacterium avium* complex (MAC). In addition, mycolic acid and fatty acid chromatography patterns are very similar to those of *Mycobacterium simiae* [1]. Thus, the identification of *M. lentiflavum* is usually confirmed through genetic analysis. Because like other NTM, *M. lentiflavum* is often isolated fortuitously, the clinical significance must be carefully considered.

Recently several cases of human diseases caused by *M. lentiflavum* have been reported, including cervical lymphadenitis, fatal disseminated infection, ascites, soft tissue, liver abscess, and pulmonary infection in immunocompetent and immunosuppressed patients [2–12]. However, there are few reports on *M. lentiflavum* as a cause of pulmonary disease and to date it has rarely been described in immunocompetent patients.

Herein, we present a case of acute necrotizing pneumonia combined with parapneumonic effusion caused by *M. lentiflavum* in an immunocompetent patient.

## Case presentation

**Fig. 1:**
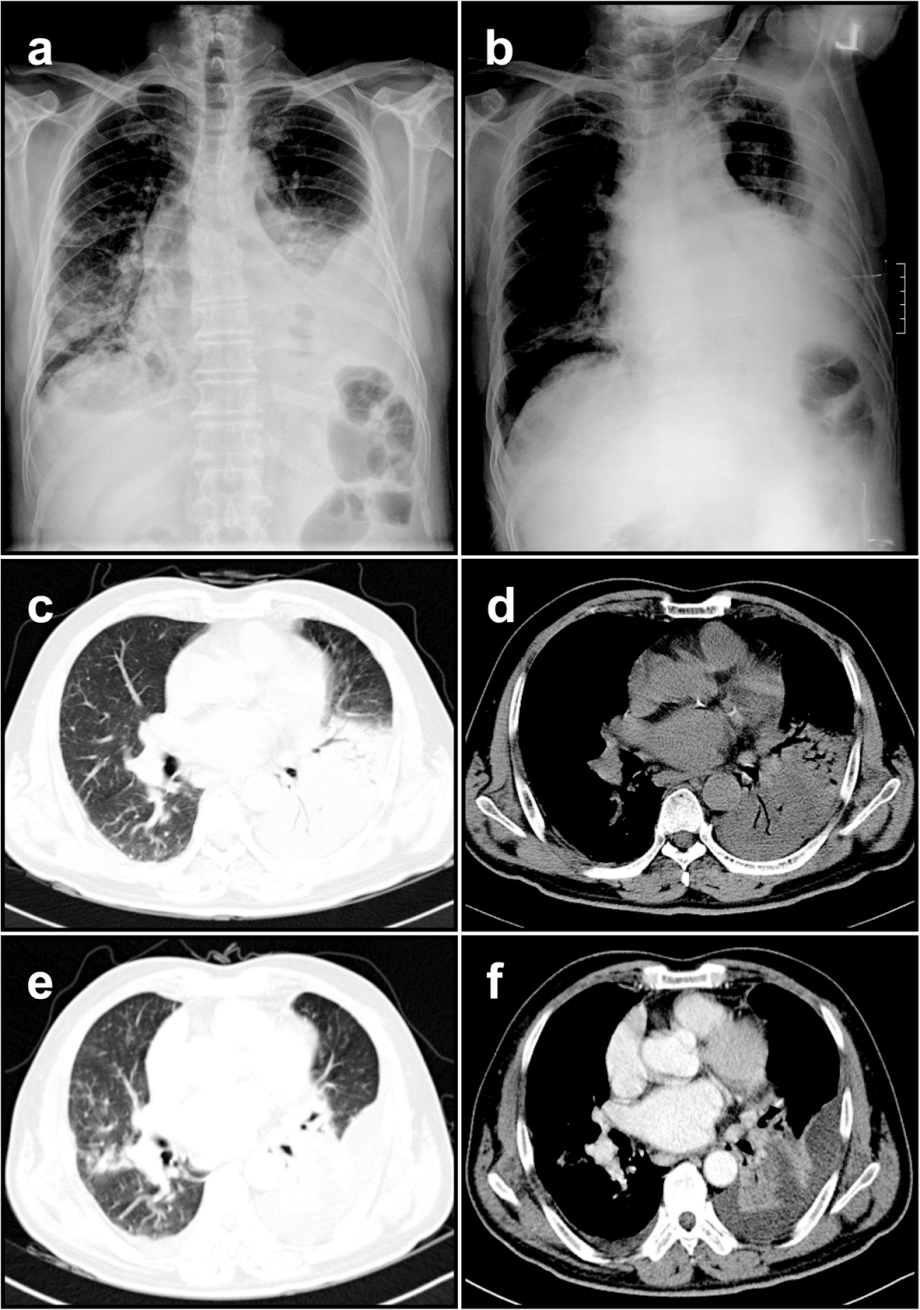
Chest X-ray and chest computed tomography (CT) findings in *M. lentiflavum*-infected patient. Chest Radiography (**a**; posteroanterior view, **b**; left decubitus view) shows consolidative lesion in the left lower lobe with fluid shifting sign. Chest CT scan revealed that solid consolidative lesion occupied the left lower lobe with air-bronchogram (**c** and **d**). Despite of the antibiotics therapy, the radiologic features were aggravated showing abscess formation in the left lower lobe, accompanying left pleural effusion and newly developed pneumonic infiltrative lesion on the right middle lobe (**e** and **f**)

A 60-year-old man was admitted to our hospital with fever, chills, productive cough, and dyspnea for last two weeks, which had exacerbated for preceding three days despite taking oral antibiotics for about 10 days. Since he had been diagnosed to have bronchial asthma three years ago, he had taken the combined inhaled medicine of corticosteroid and a long-acting β_2_ agonist. However, he had no previous history of structural lung diseases including pulmonary tuberculosis and immunocompromised disorders such as HIV infection. He was an ex-smoker with 20 pack/year smoking history and his occupation was a farmer. On physical examination, inspiratory crackles were heard mainly on the left middle and lower lung fields. Chest radiography showed pulmonary consolidation with air bronchogram in the left lower lobe, which also showed fluid shifting sign (Fig. 1a and b). Chest computed tomography (CT) scan revealed bronchopneumonic infiltration with abscess formation in the left lower lobe, accompanying left pleural effusion (Fig. 1c and d). Bacteriologic examinations including Gram stain with sputum, pleural fluids, and bronchial washing fluids revealed no definitive causative organisms. Pleural tissues were also obtained through needle biopsy, in which the acute suppurative pleural inflammatory reaction was found and some tissues were cultured to examine any bacterial or fungal growth. As a result, there was no evidence of growth of bacteria or fungi for seven days they had been cultured.

**Fig. 2:**
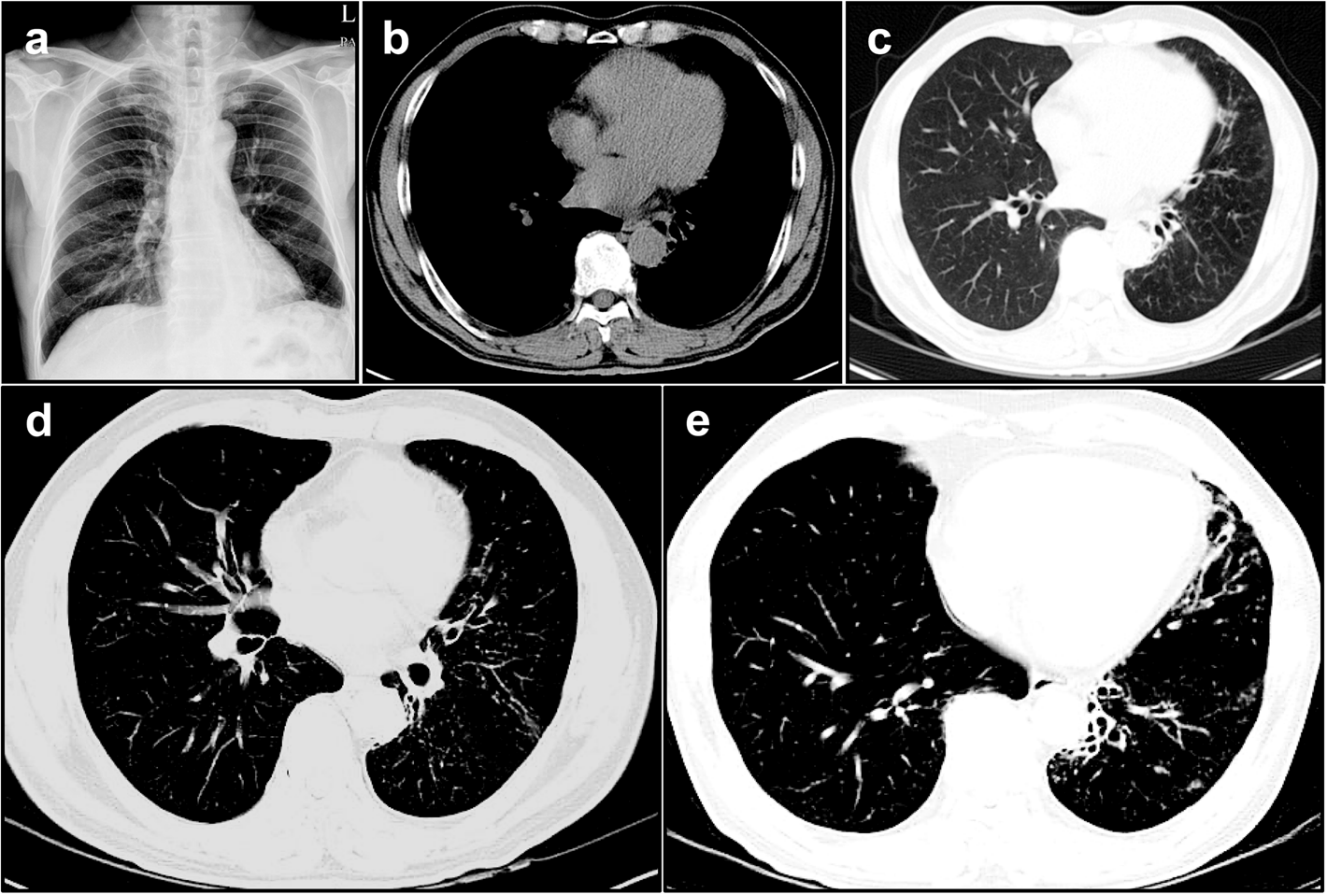
Serial chest imaging findings in *M. lentiflavum*-infected patient. At 12 month follow-up, chest imaging studies revealed that the previous lesions in both lungs were improved remarkably (**a**-**c**). In addition, serial chest CT scans showed the distinct bronchiectasis and multiple small nodular lesions with a tree-in-bud pattern were scattered in the left lingular segment and the left lower lobe (**d** and **e**)

Firstly, levofloxacin was prescribed as a single intravenous regimen. Up to hospital day 3, however, his clinical manifestation did not respond to the antibiotic therapy and even worsened (Fig. 1e and f). We decided to change the antibiotic regimen to aztreonam and metronidazole as a combination regimen. On hospital day 7, his symptoms including fever started to be resolved and then followed by radiologic improvement on chest radiography. After his discharge, we got a final bacteriologic report describing that *M. lentiflavum* was isolated from pleural specimen biopsied. The antibiotic regimen covering NTM (etambutol, ciprofloxacin, rifabutin, and clarithromycin) was prescribed to him in out-patient department, and his remnant lesions kept being improved (Fig. 2a-c), showing bronchiectasis and multiple smaller nodules of a tree-in-bud pattern in the left lingular segment and the left lower lobe (Fig. 2d and e).

## Discussion

In 1959, Runyon proposed the first classification system of mycobacteria in which, based on growth rates, colony morphology, and pigmentation in the presence and absence of light, mycobacteria were divided into four groups of human pathogens; *M. tuberculosis* complex, *M. leprae*, slowly growing NTM, and rapidly growing NTM [13]. The NTM are usually acquired from environmental sources such as surface water, tap water, soil, domestic and wild animals, milk, and food products. Though most species are less pathogenic or sporadic, they can, in broad terms, induce four distinct clinical syndromes [14]; 1) Progressive pulmonary disease, especially in older persons with or without underlying lung disease. 2) Superficial lymphadenitis, especially cervical lymphadenitis. 3) Disseminated disease in severely immunocompromised patients. 4) Skin and soft tissue infection usually as a consequence of direct inoculation.

Recently, with the availability of 16S ribosomal RNA sequencing and high-performance liquid chromatography, and polymerase chain reaction-restriction length polymorphism analysis, the number of new species of NTM including *M. lentiflavum* has risen dramatically [14, 15]. In fact, *M. lentiflavum*, one of slowly growing NTM, was first identified in a cluster of 22 isolates [1]. Among them, only one isolate from a vertebral disk in an elderly patient suffering from spondilodiscitis was clinically significant. Since then, several cases of isolates from cervical lymphadenitis of very young children have been reported [2–8]. Other infected sites including pulmonary infection are less frequent [1, 4, 6–12].

Typically, pulmonary disease by NTM is characterized as chronic progressive infectious one which develops both in healthy persons and in those with underlying pulmonary disorders or immunosuppressive conditions. The clinical features may resemble slowly progressive pulmonary tuberculosis, which is often the initial diagnosis in patients with positive results on acid fast bacilli (AFB) staining. Radiologic manifestations of pulmonary infection with NTM are various, to some degree, non-specific including the formation of solitary or multiple nodules, chronic pneumonitis, bronchiectasis, cavitary formation, or a combination of these features.

Altogether, the diagnosis of pulmonary infection with NTM is extremely difficult due to several factors involved; these are various clinical and radiologic manifestations, the frequent presence of significant prior pulmonary disease, and harmless colonization of NTM in the lower respiratory tracts. According to American Thoracic Society (ATS)/ Infectious Diseases Society of America (IDSA) guidelines, the diagnosis should be based on specific, validated criteria that emphasize a compatible clinical syndrome, characteristic findings on chest X-ray or CT, and repeated isolation of NTM from the sputum or growth of NTM from a lung biopsy [16]. Thus, in cases with these typical radiologic findings but with negative results of routine sputum cultures for mycobacteria, physicians have been subsequently recommended to perform bronchoscopy and transbronchial biopsy.

To our knowledge, this report is the first case of acute necrotizing pneumonia with pleurisy by *M. lentiflavum* in an immunocompetent patient without underlying pulmonary structural disorders. In our case, a 60-year-old man having prolonged productive cough and dyspnea with fever was initially diagnosed as pneumonia with parapneumonic effusion. Imaging studies showed that the radiologic abnormality was acute bronchopneumonic infiltration with abscess formation in the left lower lobe and parapneumonic pleural effusion. Bacteriologic examinations with sputum, pleural fluids, and bronchial washing fluids, however, revealed no definitive causative organisms. Fortunately, we could confirm that the etiologic organism was *M. lentiflavum* found in the cultured pleural tissues, although there is still a possibility of co-infection with other bacteria such as anaerobes because it is difficult to culture or isolate anaerobes in several respiratory samples and the patient was pre-treated with the antibiotics that would have negated bacterial cultures or have activity against co-infected bacteria. On the basis of these findings, he was diagnosed as pulmonary infection and pleurisy caused by *M. Lentiflavum* as at least one of etiological pathogens, which was treated with a combination of antibiotics covering NTM.

## Conclusions

In light of general information of NTM, this case report describes several interesting points as follows; the acute progress of NTM pulmonary diseases, pleural disease by NTM infection, and occurence of pulmonary infection by *M. lentiflavum* in immunocompetent subject. Thus, we suggest that clinicians should consider the *M. lentiflavum* as a human pathogen invovled in lung and pleura regardless of immune status. In addition, *M. lentiflavum* can be an infectious cause of acute pulmonary disease as well as chronic pulmonary disease.

## Consent

Written informed consent was obtained from the patient for publication of this case report. Copies of the written consents are available for review by the editor of this journal.

## Abbreviations

MAC: *Mycobacterium avium* complex
NTM: Nontuberculous mycobacterium
